# The Impact of Telehealth on Clinical Education in Adolescent Medicine During the COVID-19 Pandemic: Positive Preliminary Findings

**DOI:** 10.3389/fped.2021.642279

**Published:** 2021-03-19

**Authors:** Do-Quyen Pham, Sarah A. Golub, Cora Collette Breuner, Yolanda N. Evans

**Affiliations:** ^1^Division of Adolescent Medicine, Seattle Children's Hospital, Seattle, WA, United States; ^2^Department of Pediatrics, University of Washington School of Medicine, Seattle, WA, United States

**Keywords:** telehealth, COVID-19, pandemic, adolescent medicine, clinical education, medical education

## Abstract

**Purpose:** Following the start of the COVID-19 pandemic, much of clinical care rapidly transitioned to telehealth, shifting the clinical training milieu for most trainees. In the wake of this shift, educators have attempted to keep learners engaged in patient care and optimize medical education as much as possible. There is, however, limited understanding of the effect of telehealth on clinical education. The aim of our study was to better understand the educational experience of pediatric and Adolescent Medicine trainees participating in clinical encounters via telehealth in a specialty consultation Adolescent Medicine Clinic at a quaternary pediatric care hospital.

**Methods:** Using a web-based anonymous questionnaire, we surveyed trainees rotating through the Adolescent Medicine Clinic between March and June 2020. We used descriptive statistics to evaluate their experiences with telehealth and identify techniques that were effective to facilitate learning during a telehealth visit.

**Results:** Surveys from 12 pediatric and Adolescent Medicine trainees were received, a 75% response rate. Most trainees (83.3%) reported no prior experience with telehealth before the onset of the pandemic. By the end of their rotation, trainees identified techniques that helped facilitate learning during a telehealth visit. The majority of trainees (83.3%) rated their experience as effective or very effective, and all reported interest in incorporating telehealth into their future practice.

**Conclusions:** Pediatric and Adolescent Medicine trainees reported overall positive experiences with telehealth in clinical education and an interest in incorporating this tool into future practice. Additional research is needed to refine techniques in engaging learners through telehealth.

## Introduction

The onset of the COVID-19 pandemic propelled into motion a rapid transition of many aspects of society across the globe, as life as we once knew it transformed over a period of weeks. While telehealth has been a well-established means of providing clinical services for over a decade, the global pandemic has brought it to the forefront as a crucial means of providing continued access to care while minimizing disease transmission (1, 2).

Existing literature has shown that many components of adolescent health care can be provided effectively through telehealth (3–5). In the past year, several academic institutions in the United States have also published on the challenges, opportunities, and impact of a rapid scale-up of telehealth use in Adolescent Medicine in the setting of the pandemic (6–9). However, with academic medical centers worldwide converting much of their clinical care to telehealth, the educational opportunities and roles of medical trainees have shifted as well. Given the extraordinary nature of the pandemic, there have been varied approaches to managing trainees, ranging from complete discouragement of participation in clinical care to throwing trainees into action or fast-tracking students to graduate early to enable their participation in clinical care (10).

Keeping trainees engaged and on the frontline is viewed by some as a great teaching opportunity despite the fact that their participation potentially increases their exposure to disease. Many would argue that it promotes critical thinking skills essential for growth. On the other hand, trainees left at home on the sideline may feel excluded, wondering how they could meaningfully contribute to clinical care and further their education (11). Telehealth, however, has thus far proven to be a solution in bridging this gap, enabling medical trainees to safely observe and participate in patient care remotely during the COVID-19 pandemic (12). In some cases, medical trainees have also served as “digital natives” and helped play a role in the expansion and delivery of healthcare in the setting of COVID-19 (10). Through various frameworks, medical educators have proposed methods to ensure inclusion of learners; however, balancing safety for patients and staff, while keeping trainees engaged, has proven to be a difficult task (13).

Despite the growing body of relevant literature describing the importance of including learners in the virtual setting, there is a dearth of evidence regarding the efficacy of the telehealth platform in how it specifically affects clinical education. In addition, many of these publications have focused on medical student education and not on residents or clinical fellows, and none have explored Adolescent Medicine trainees' perspectives (1, 14–16). The aim of our study was to assess the educational experience of pediatric and Adolescent Medicine trainees participating in clinical encounters via telehealth in a specialty Adolescent Medicine Clinic.

## Methods

We developed a brief, web-based survey to gather responses from residents and fellows rotating through the Adolescent Medicine Clinic at Seattle Children's Hospital (SCH). Participants included pediatric residents in their 3rd year of training during a required 4-week rotation concentrated on adolescent health. In addition, Adolescent Medicine fellows who had previously completed residency training prior to pursuing subspecialty training in Adolescent Medicine were also included. We have 3–4 Adolescent Medicine fellows and 2–4 pediatric residents working in our clinic each month. This clinic provides multiple consultative services including gender-affirming care, eating disorders, reproductive healthcare, behavioral health services, and biofeedback therapy. Each clinic half-day session is 4 h. The setting includes unique educational opportunities to participate in multidisciplinary care while navigating adolescent confidentiality.

This anonymous questionnaire consisted of 11 discrete items which elicited learners' feedback regarding their experiences using telemedicine. Items included multiple choice, Likert-scale, and free-text responses. The survey was administered between March and June 2020. Initial request to complete the survey was sent out by email, followed by a reminder email 1 week later. We used simple descriptive statistics to analyze quantitative survey results. The current paper presents the quantitative component of a mixed-methods study; free-text responses were analyzed qualitatively and were described in our previous work. We received approval from the SCH Institutional Review Board (IRB) to conduct this study.

## Results

We received completed surveys from 12 trainees (a 75% response rate) that consisted of 9 third-year pediatric residents and 3 Adolescent Medicine fellows. At the time of survey administration, our Division supported 4 clinical fellows, and up to 4 pediatric residents per 4-week block. Table 1 summarizes participants' level of training, post-graduate career plans, and experience with telehealth prior to and after the onset of the pandemic. Prior to the rotation, <20% of trainees had previous experience with telehealth. By the end of the study, the majority of trainees (66.7%) had attended more than 10 telehealth clinic sessions.

**Table 1:** Characteristics of trainees and experience with telehealth.

	***N* (%)**
**Level of training**	
Pediatric resident	9 (75)
Fellow	3 (25)
Post-graduate career plans^*^	
Primary care	5 (41.7)
Subspecialty care	6 (50)
Hospital medicine	2 (16.7)
Career in research	1 (8.3)
**Previous experience with telehealth**	
Yes	2 (16.7)
No	10 (83.3)
Number of telehealth clinic sessions attended^**^	
1–5	3 (25)
6–10	1 (8.3)
11–15	5 (41.7)
>15	3 (25)

* *Categories are not mutually exclusive*.

** *Refers to the total number of sessions attended in the Adolescent Medicine clinic. One clinic session refers to a half-day of clinic (4 h)*.

**Figure 1:**
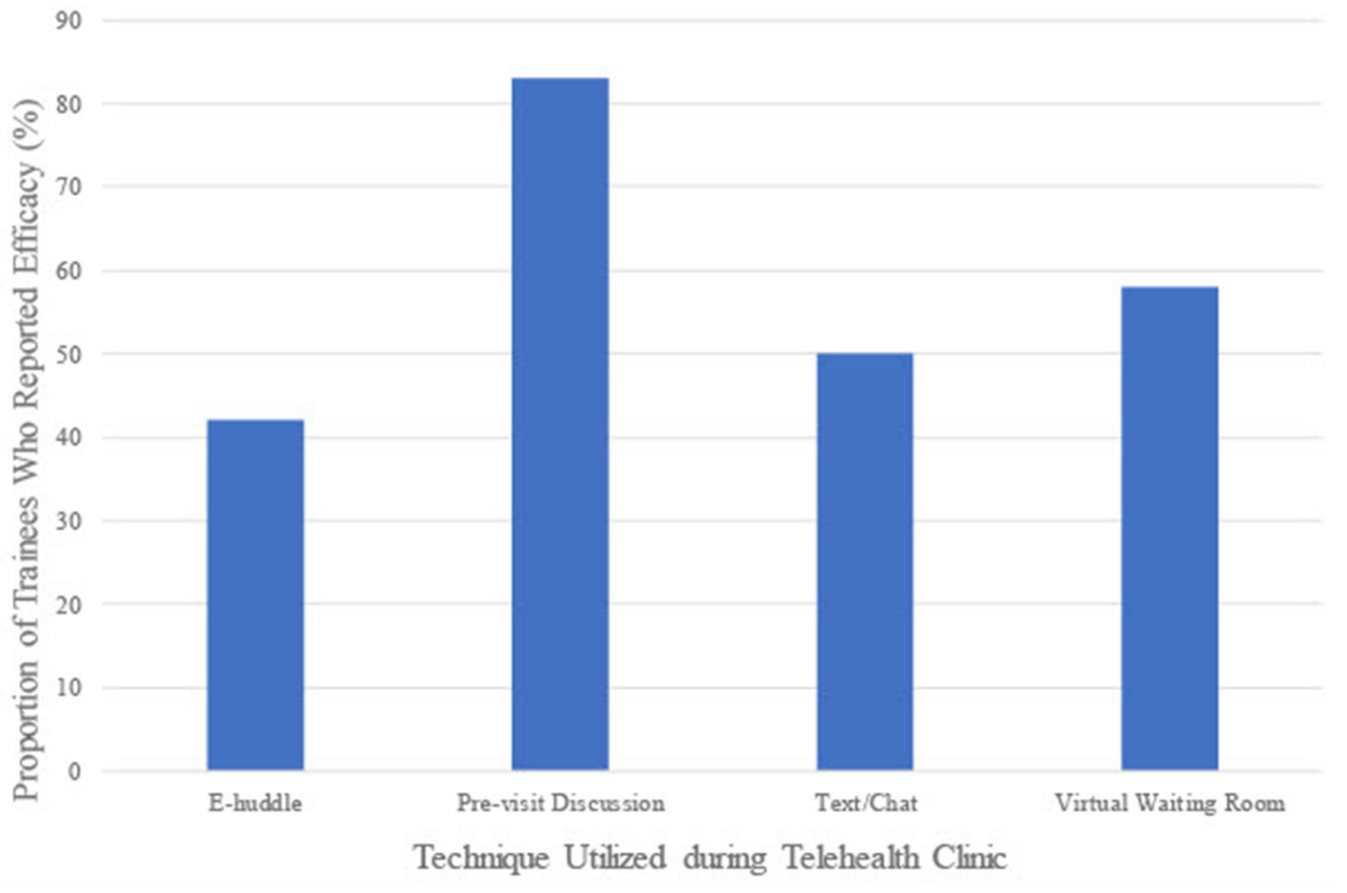
Effective techniques to facilitate clinical education through telehealth.

Participants identified several techniques, some of which were unique to the telehealth setting, that facilitated their clinical education during the rotation (Figure 1). Secure texting or private chat function during the appointment, where a trainee and attending physician sent direct messages to one another through the telehealth platform, was identified by 50% of trainees to be effective. Moving a patient to a virtual waiting room in the telehealth platform to facilitate direct discussion between the preceptor and trainee was reported by 58.3% of trainees to be effective. Many attendings also sent an electronic huddle (hereafter referred to as an e-huddle) to trainees and clinical staff prior to clinic. E-huddles allow attendings to provide a brief summary of patients scheduled for the purpose of designating clinical tasks to medical assistants, nurses and social workers, and to outline a presumptive plan for each patient. Attendings email these huddles to appropriate members of the clinic team and their assigned trainee prior to their scheduled clinics in order to facilitate patient care. Fellows were responsible for preparing and sending their own e-huddles, as patients are scheduled on their own clinic templates (while residents see patients on the attending physician's template). Although not unique to the telehealth platform, e-huddles sent out by the preceptor and pre-visit case discussions with preceptor were additional ways to facilitate clinical education through telehealth. E-huddles and pre-visit case discussions were reported as effective by 41.7% and 83.3% of trainees, respectively. Pre-visit case discussions were opportunities where the attending and trainee could discuss a clinical case prior to the medical visit and facilitated direct conversations between the attending and trainee. When reviewing perceived efficacy of e-huddles reported by residents and fellows separately, a greater proportion of residents (66.7%) reported this tool as effective compared to fellows (0%).

The majority of trainees (83.3%) rated their experience with telehealth as either effective or very effective. Among those who had more than 5 telehealth sessions, all reported that telehealth was effective or very effective in clinical education. All 12 trainees, regardless of frequency of telehealth clinics, reported interest in incorporating telehealth into their future practice.

## Discussion

Despite the abrupt changes brought on by the pandemic and the swift transition to telehealth, our study suggests that trainees have found telehealth to be an effective and positive aspect of their clinical education and training in the Adolescent Medicine Clinic. This study expands on our previous work, which included a qualitative analysis exploring Adolescent Medicine trainees' perspectives on the educational impact of telehealth (work in submission). The current manuscript adds by identifying and evaluating the utility of specific educational techniques that have been employed to enhance the clinical experience for trainees.

In the months since the onset of the pandemic, literature on the myriad challenges of medical education during COVID-19 has increased, though few prior studies have explored or targeted specific strategies used for clinical teaching in the virtual space. Of these studies, the vast majority have focused on education not involving direct patient care. Theoret et al. mentions the concept of a virtual anatomy dissection for medical students, by means of utilizing the screen-sharing function (17). In addition, Dedeilia et al. reviews various innovations for surgical and medical trainees including flipped online learning classrooms, simulation models and even oral examinations performed via teleconferences (18). Chick et al. outlines in detail the precepting model used in telehealth clinics for surgical residents, with trainees initially gathering history, formulating a plan, and then reviewing the case with the attending surgeon by phone before a final wrap-up via video conference, followed by a post-visit discussion of the case after the patient has signed off (19). Pourmand et al. also suggests that in considering future curricula involving telemedicine for medical trainees, it may be important to develop a consensus of which skills or milestones should be required at each stage of training (20). None of these studies, however, provide recommendations or feedback regarding mechanisms to support trainee education through telehealth encounters. In addition, to our knowledge, at the time of this manuscript preparation, there were no studies focusing on the educational experience of telehealth from the perspective of trainees in a pediatric hospital, or trainees caring for adolescents.

Strengths of the study include a high response rate of 75%. Additionally, responses were gathered from trainees shortly after the completion of their rotation, limiting recall bias. Given the anonymous nature of the survey and the fact that responses were elicited after completion of the rotation, there was little concern for trainees feeling influenced by faculty when providing their feedback. Our study has several limitations, one of which is the small sample size of trainees. In addition, given that supervising providers were learning and adapting to a relatively new modality of healthcare delivery, the educational experiences of trainees may have varied over time; it is likely that trainees rotating in June may have had a different educational experience than those rotating in March, at the very start of the pandemic. As healthcare providers and institutions continue to refine delivery of healthcare in the virtual setting, we anticipate that these improvements will allow preceptors to continue to enhance their teaching skills and thus, further education of trainees in this setting as well.

This small study was carried out with the goal of hypothesis development for further larger-scale studies of similar nature in the future. In the months since initial survey administration, in efforts to limit disease transmission, our Division has begun to expand telehealth services in order to reach vulnerable youth. Examples of these settings include juvenile detention center, transitional housing centers, and emergency shelters for adolescents experiencing homelessness. We aim to consistently involve learners in these areas and subsequently hope to evaluate the quality of their educational experiences in these unique spaces. While this preliminary study has identified some tools and strategies to provide clinical education via telehealth, more research is needed to refine these techniques.

In summary, our study implies that with utilization of appropriate teaching strategies, telehealth can be a valuable tool in the clinical education of pediatric and Adolescent Medicine trainees. The abrupt transformation to widespread use of telehealth in clinical care has forced educators to reexamine their approaches to clinical teaching, while upholding their duty to support trainees during this unprecedented time in history. While disruptive, this forced overhaul of medical curricula may push us to explore novel approaches to education to benefit the next generation of medical providers.

## Data Availability Statement

The raw data supporting the conclusions of this article will be made available by the authors, without undue reservation.

## Ethics Statement

The studies involving human participants were reviewed and approved by Seattle Children's Hospital Institutional Review Board (IRB). The patients/participants provided their written informed consent to participate in this study.

## Author Contributions

D-QP and SG were responsible for study design, survey development, data analysis, and manuscript preparation. CB and YE were responsible for study design, survey development, and manuscript editing. All authors contributed to the article and approved the submitted version.

## Conflict of Interest

The authors declare that the research was conducted in the absence of any commercial or financial relationships that could be construed as a potential conflict of interest.
